# An Indirect Action Contributes to C-Fos Induction in Paraventricular Hypothalamic Nucleus by Neuropeptide Y

**DOI:** 10.1038/srep19980

**Published:** 2016-01-27

**Authors:** Shengjie Fan, Janani Dakshinamoorthy, Eun Ran Kim, Yong Xu, Cheng Huang, Qingchun Tong

**Affiliations:** 1 School of Pharmacy, Shanghai University of Traditional Chinese Medicine, 1200 Cailun Road, Shanghai 201203, China; 2 Brown Foundation Institute of Molecular Medicine and Program in Neuroscience, Graduate School of Biological Sciences, University of Texas McGovern Medical School, Houston, TX 77030 USA; 3 Children’s Nutrition Research Center, Department of Pediatrics, Baylor College of Medicine, One Baylor Plaza, Houston, TX 77030, USA

## Abstract

Neuropeptide Y (NPY) is a well-established orexigenic peptide and hypothalamic paraventricular nucleus (PVH) is one major brain site that mediates the orexigenic action of NPY. NPY induces abundant expression of C-Fos, an indicator for neuronal activation, in the PVH, which has been used extensively to examine the underlying NPY orexigenic neural pathways. However, PVH C-Fos induction is in discordance with the abundant expression of NPY receptors, a group of inhibitory Gi protein coupled receptors in the PVH, and with the overall role of PVH neurons in feeding inhibition, suggesting a mechanism of indirect action. Here we showed that the ability of NPY on C-Fos induction in the PVH was blunted in conditions of insulin deficiency and fasting, a condition associated with a high level of NPY and a low level of insulin. Moreover, insulin insufficiency blunted C-Fos induction in the PVH by fasting-induced re-feeding, and insulin and NPY induced c-Fos induction in the same group of PVH neurons. Finally, NPY produced normal C-Fos induction in the PVH with disruption of GABA-A receptors. Thus, our results revealed that PVH C-Fos induction by NPY is mediated by an indirect action, which is at least partially mediated by insulin action, but not GABA-A receptors.

Neuropeptide Y (NPY) is one of the most potent endogenous orexigenic peptides^1^,^2^. Although broadly expressed in scattered neurons in the brain, NPY shows abundant expression in the arcuate nucleus (Arc) of the hypothalamus, where it is exclusively located in agouti-related protein (AgRP) neurons^3^, a group of neurons critical for feeding regulation. AgRP/NPY neurons project to several brain sites, including the paraventricular nucleus of the hypothalamus (PVH), another key brain region in feeding regulation, which receives intensive projections from AgRP neurons^4^. Direct release of GABA and NPY from AgRP neurons to PVH neurons elicits feeding^4^,^5^, suggesting a role for NPY action on PVH neurons in feeding regulation. Consistently, PVH expresses NPY Y1 and Y5 receptors^6^,^7^, two NPY receptor isoforms known to be most relevant to the NPY feeding behavior. However, despite extensive research on NPY feeding regulation, the neural pathway that mediates the NPY action is not yet clear.

One of key features associated with NPY-induced feeding is the strong induction of the expression of *c-fos*^8^,^9^, an immediate early response gene for neuron activation, in several feeding-related brain regions, including PVH. The C-Fos induction in the PVH has been used extensively to examine the NPY feeding pathway and can be reversed by antagonists of NPY Y1 and Y5 receptors^8^,^10^,^11^,^12^, implying a direct action on PVH neurons However, despite the demonstrated importance of AgRP/NPY neuronal projections to the PVH in feeding regulation, the mechanism responsible for NPY-induced C-Fos in the PVH is not clear. Of note, the fact that NPY induces strong C-Fos induction in the PVH is in apparent contradiction with the known neural pathway involving NPY. For example, lesion of the PVH causes hyperphagia and specific activation of PVH neurons leads to reduced feeding^13^,^14^,^15^, suggesting an overall role for PVH neurons in feeding inhibition. The expression of C-Fos by NPY, an indicator for an increased level of neuron activity of PVH neurons, contradicts with NPY-induced hyperphagic effects. Substantial evidence suggests that AgRP/NPY neurons send mono-synaptic projections to PVH neurons^4^,^16^. Consistent with this, adult lesion of AgRP neurons caused robust C-Fos expression in the PVH^7^,^17^,^18^, suggesting disinhibition due to removal of AgRP neuron projection. Since AgRP neuron lesion also causes NPY elimination in the Arc, this C-Fos up-regulation is in contradiction with NPY-induced c-Fos in the same brain region. Importantly, NPY receptors Y1 and Y5 are inhibitory Gi protein coupled receptors^7^,^18^,^19^, which also contradicts with direct effect of NPY on the induction of C-Fos in the PVH. Moreover, previous electrophysiological studies suggest that NPY either directly inhibits PVH neurons or reduces GABAergic inputs to these neurons^20^,^21^,^22^. These observations, taken together, suggest that the C-Fos induction by NPY in the PVH may be mediated through an indirect action. However, the nature of this indirection action is unknown.

Earlier studies demonstrated that brain NPY action increases blood insulin levels^23^,^24^,^25^. Recent studies suggest that insulin is capable of effectively modulating neuron activity, raising a possibility for insulin action in mediating NPY induction of C-Fos in the PVH. In addition, GABA-A receptors in the PVH have also been suggested to mediate NPY action on modulating PVH neuron activity and feeding^20^,^26^, also raising a possibility for PVH GABA-A receptors in mediating NPY induction of C-Fos. Thus, in the current study, we examined whether insulin action and PVH GABA-A receptors contribute to NPY induced C-Fos in the PVH.

## Material and Methods

### Animals

Male wild-type mice, *Sim1-Cre:γ2*^*flox/flox*^ and their littermate control mice were maintained in a temperature (22 ± 1 °C) and light (12:12 h light dark cycle, lights on at 7 pm and off at 7 pm) controlled environment with water and regular chow food provided ad libitum. Animal care and procedures were performed in accordance with relevant guidelines and regulations and approved by the University of Texas Health Science Center at Houston Institutional Animal Care and Use Committee. Generation of *Sim1-Cre:γ2*^*flox/flox*^ mice was described previously^27^.

### STZ-induced type 1 diabetes

Type 1 diabetes mellitus was induced in 7-week-old male mice by a single intraperitoneal (i.p.) injection of streptozotocin (STZ, 150 mg/kg body weight)^28^. Body weight- and age-matched male mice injected with the same volume of saline were used as controls. STZ-treated mice with a sustained blood glucose over 500 mg/dl for more than 3 consecutive days measured 1 week post-injections were qualified as being type 1 diabetic.

### Intracerebroventricular (icv) cannulation and drug administration

Surgery and intracerebral injections were performed as described previously^27^. Briefly, male mice (7–10 weeks of age) were anesthetized with Ketamine/Xylazine (100 mg/kg, 10 mg/kg, i.p.) and placed in a stereotaxic frame (David Kopf Instruments). Guide cannula (26 gauge, 5 mm, Plastics One, VA, USA) were inserted into right lateral brain ventricle with a coordinate (0.3 mm posterior to the bregma, 1 mm to the right from the midline and 2.5 mm deep to the superior surface of the skull), and were secured to skull with dental acrylic cement. After a minimum 7-day recovery, mice were tested with angiotensin to confirm i.c.v injection would be successful. Intensive drinking behavior immediately after angiotensin injection through the implanted cannula was used as an indication of successful implantation of cannula. For experiments, saline (2 μl), NPY (1 mg/ml) or insulin (5 mU/μl) was slowly administered through the guide cannula.

### Feeding and hormone measurements

Food intake was measured as the amount of food consumed during a 2-hour period after NPY injections or feeding initiation after overnight fasting. For non-fasting sections, food was withdrawn for 2 hours before measurement of feeding in the morning. Insulin was measured in tail blood from STZ-treated and control mice by the Vanderbilt Hormonal Core Facility.

### Immunohistochemistry assays

Mice were anesthetized with Ketamine/Xylazine (100 mg/kg, 10 mg/kg, i.p.) and perfused transcardially with saline and formalin subsequently. Brains and pancreas tissues were post-fixed in formalin overnight and immersed in 30% sucrose in 0.01 M PBS at 4 °C. A series of 30-μm thick coronal brain sections were cut on a microtome (Leica SM 2010R, Germany) and were used for immunostaining C-Fos. After OCT embedding, the pancreas was cut into 7-μm thick sections on a cryostat (Leica CM 1850, Germany) for immunostaining insulin and glucagon.

Immunohistochemistry (IHC) was performed as previously described^29^. Pancreas sections were double stained with rabbit anti-glucagon (1:500, Cell Signaling Technology, MA, USA) and mouse anti-insulin (1:400, Thermo Scientific, CA, USA) antibodies, and were visualized with Alexa fluor 488- or Alexa fluor 594- labelled second antibodies (1:200, Jackson ImmunoResarch, PA, USA), respectively.

To visualize *c-fos* expression in PVH, mice were perfused 2 h post NPY-, saline- or insulin- injection or fasted-refed treatment. Brain sections were then incubated with rabbit anti-C-Fos (1:1000; Millipore, MA, USA) antibody. C-Fos positive signals were visualized with Alexa fluor 488 conjugated donkey anti-rabbit IgG (1:200; Invitrogen, CA, USA) and were captured with a TCS SP5 confocal microscope (Leica, Nussloch, Germany).

To quantify C-Fos expression, three matched brain sections containing PVH from each mouse were chosen. The number of all immune-positive neurons in the PVH with clear profile from each section was counted and the average of C-Fos neuron number over the three sections was used as a representative number of C-Fos responsive neurons in each mouse.

### Statistics

Data sets were presented at mean ± SEM and analyzed for statistical significance using PRISM 5 (GraphPad, CA, USA) for two-tailed unpaired Student’s t-tests, or for ANOVA tests using Tukey’s multiple comparison tests. A P-value of <0.05 was required for significance.

## Results

**Figure 1:**
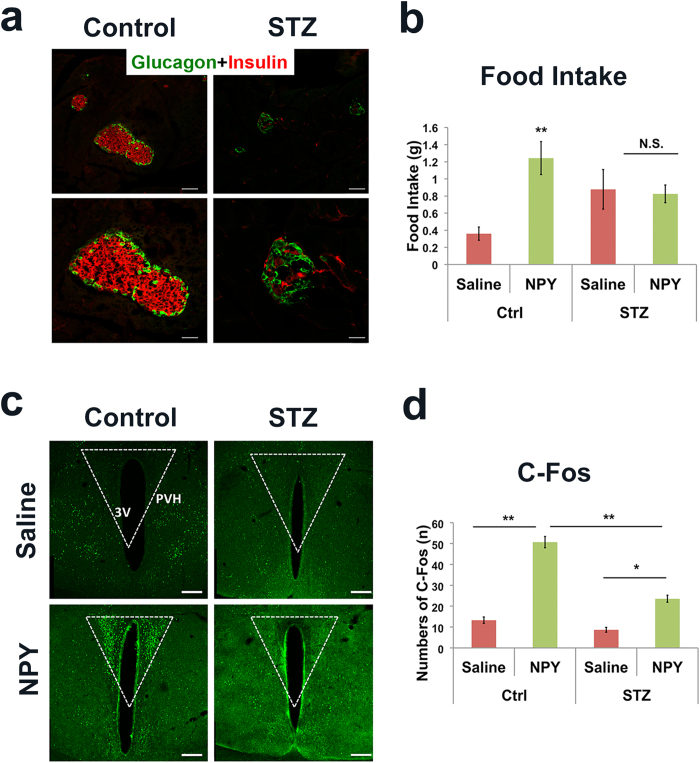
Insulin deficiency reduced NPY-induced C-Fos in the PVH. STZ-treated (150 mg/kg) insulin deficient mice and controls were icv administered with saline or NPY and C-Fos expression in the PVH was examined 2 hours later. (**a**) Representative pictures showing insulin (Red)- and glucagon (green)-immunoreative structures in pancreatic islets from controls (left panels) and insulin deficient mice (right panels). Bottom panels showed an islet with an amplified field (40×). (**b**) Food intake in both groups within the 2-hour testing period in response to saline or NPY treatment. (**c**) Representative pictures showing C-Fos immunoreactive neurons in the PVH of controls (upper panels), insulin deficient mice (bottom panels), saline treatment (left panels) and NPY treatment (right panels, 2 μg in 2 μl). (**d**) Statistical analysis of the number of C-Fos neurons in the PVH of mice described in (**c**). PVH: paraventricular hypothalamic nucleus; 3 V: the third ventricle. Scale bar: 100 μm in upper panels of (**a**), 25 μm in bottom panels of (**a**), and 500 μm in (**c**). Data presented as mean ± SEM, n = 5–6, *p < 0.05 and **p < 0.01, two way ANOVA tests with tukey post hoc analyses.

To examine whether insulin action is required for NPY to induce C-Fos, we generated mice with insulin deficiency using STZ treatment, a well-established method for pancreatic β-cell lesion^28^. Mice with sustained glucose levels more than 500 mg/dl for 3 consecutive days were used for studies. Consistent with severe hyperglycemia, pancreatic islets obtained from STZ-treated animals exhibited a dramatic reduction in insulin immuoreactive β-cell number, compared to controls (Fig. 1a). Interestingly, within 2 hours after icv NPY administration, while control mice showed a drastic increase in feeding, compared to saline controls, STZ-treated mice exhibited no increase in feeding compared to their saline controls (Fig. 1b). Although failure for NPY-induced feeding may be in part due to an increased basal level of feeding owing to insulin insufficiency, NPY induced a significant less amount of food consumption in STZ-treated diabetic animals, compared to non-diabetic controls (Fig. 1c), suggesting an inherent defective response to NPY-induced feeding response in STZ-treated mice. As expected, NPY induced a dramatic increase in the number of C-Fos neurons in the PVH in controls. However, the same NPY treatment induced a much less number of C-Fos neurons in STZ treated mice (Fig. 1c,d), suggesting that insulin deficiency blunted NPY-induced C-Fos expression in the PVH.

**Figure 2:**
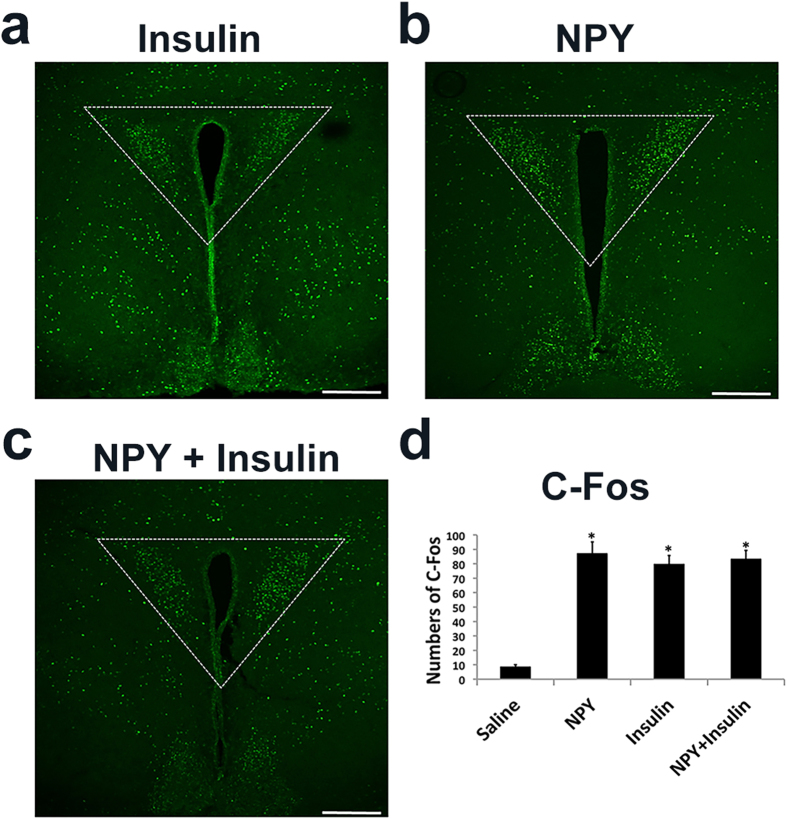
Insulin induced C-Fos expression in PVH. Wild-type mice were icv administered with saline, NPY (2 μg in 2 μl) and/or insulin (10mU in 2 μl). C-Fos expression in the PVH was examined 2 hours later. (**a**) Representative pictures showing C-Fos immunoreactive neurons in the PVH of NPY-treated mice. (**b**) C-Fos immunoreactive neurons in the PVH of insulin-treated mice. (**c**) C-Fos immunoreactive neurons in the PVH of insulin- and NPY-treated mice. (**d**) Statistical analysis of the number of C-Fos neurons in the PVH of mice described in (**a–c**). Scale bar is 500 μm. 3 V: the third ventricle. Data presented as mean ± SEM, n = 5–6 mice, **p < 0.01 vs saline, two-tailed unpaired student’s t-test.

Central NPY action induces an increase in blood insulin^23^,^24^, suggesting a possibility that NPY-induced C-Fos in the PVH is secondary to insulin action. To examine this, we directly examined C-Fos response in the brain in response to icv insulin. As expected, icv NPY induced abundant C-Fos expression in the PVH (Fig. 2a,d). Notably, insulin also induced abundant C-Fos expression, in a similar fashion to that of NPY (Fig. 2b,d). These results suggest that central insulin action is sufficient to induce C-Fos in the PVH. To examine whether insulin and NPY induce C-Fos in the same group of PVH neurons, we examined C-Fos response to simultaneous NPY and insulin injections. Interestingly, the number of C-Fos neurons in the PVH was comparable to that of insulin or NPY injection (Fig. 2c,d), suggesting that insulin and NPY induce C-Fos in the same group of PVH neurons.

**Figure 3:**
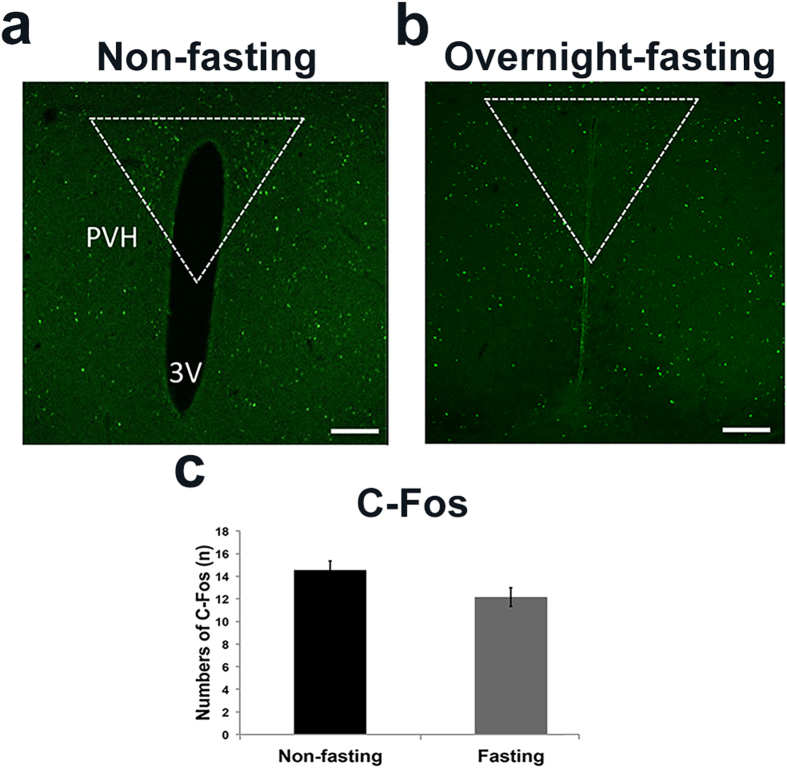
Fasting failed to induce C-Fos in PVH. Wild-type mice were well-fed or fasted overnight and C-Fos expression in the PVH was examined at the end of fasting. (**a**) Representative pictures showing C-Fos immunoreactive neurons in the PVH of non-fasted mice. (**b**) C-Fos immunoreactive neurons in the PVH of fasted mice. (**c**) Statistical analysis of the number of C-Fos neurons in the PVH of mice described in (**a,b**). 3 V: the third ventricle. Scale bar is 500 μm. Data presented as mean ± SEM, n = 5–6 mice, two-tailed unpaired student’s t-test.

If central NPY action directly induces C-Fos expression in the PVH, then conditions with high levels of NPY should be associated with higher C-Fos expression in the PVH. Fasting is known to be associated with a high level of AgRP/NPY neuron activity and a high level of NPY^3^,^30^,^31^. Thus, we compared C-Fos expression in the PVH between mice with non-fasting (Fig. 3a) and overnight fasting conditions (Fig. 3b). There was no difference in the number of C-Fos expression between the two groups of mice, suggesting that a high level of NPY doesn’t necessarily result in a high level of C-Fos expression in the PVH.

**Figure 4:**
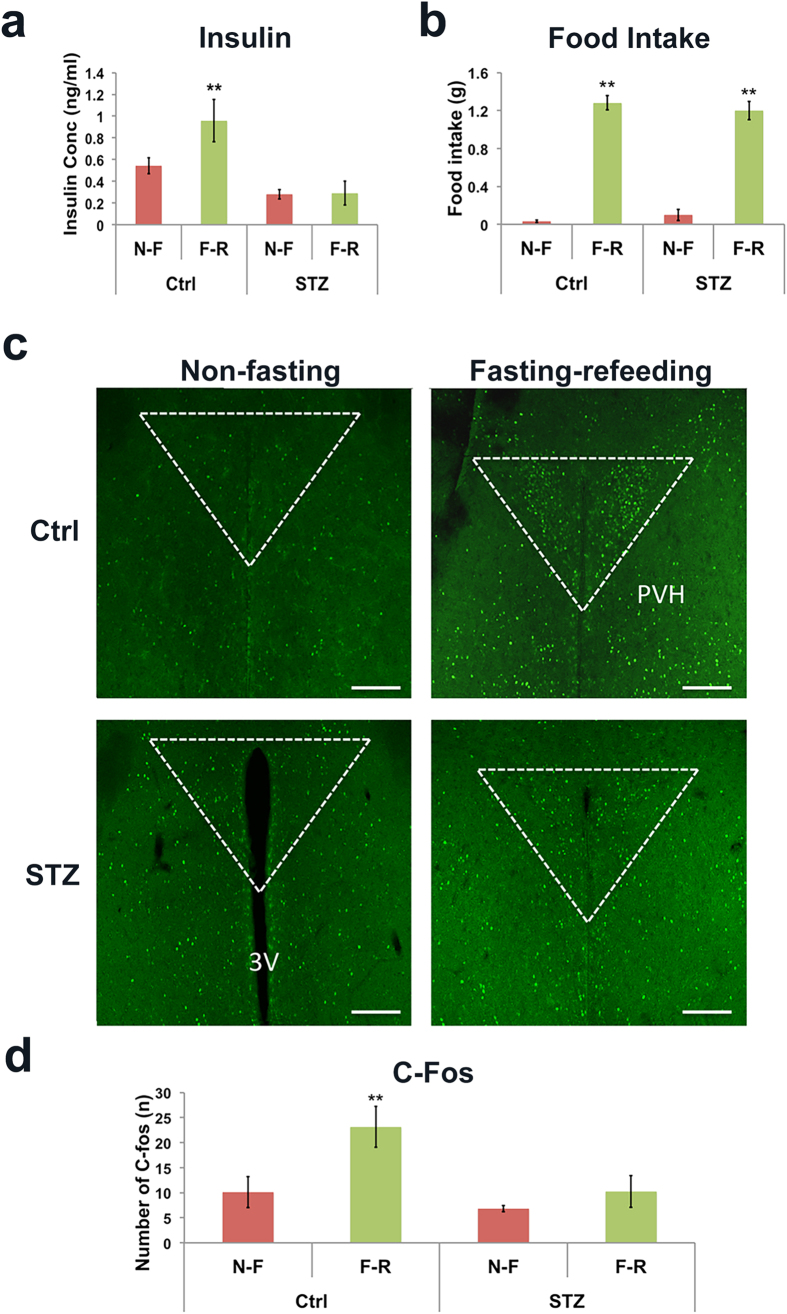
Fasting-refeeding induced C-Fos in PVH. STZ-treated (150 mg/kg) insulin deficient mice and controls were well-fed or 2 hour-refed after overnight fasting, and C-Fos expression in the PVH was examined after refeeding. (**a**) Serum insulin concentration in 4 groups after refed 15 min. (**b**) Food intake in 4 groups within the 2-hour testing period. (**c**) Representative pictures showing C-Fos immunoreactive neurons in the PVH of control (upper panels), STZ mice (bottom panels), non-fasting (left panels) and fasting-refeeding treatment (right panels). (**d**) Statistical analysis of the number of C-Fos neurons in the PVH of mice described in (**c**). Ctrl N-F: Control non-fasting mice, Ctrl F-R: Control fasting-refeeding mice, STZ N-F: STZ non-fasting mice, STZ F-R: STZ fasting-refeeding mice. Scale bar is 500 μm. 3 V: the third ventricle. Data presented as mean ± SEM, n = 5–6, **p < 0.01, two way ANOVA tests with tukey post hoc analyses.

We then examined whether conditions with physiologically increased insulin levels are associated with increased C-Fos expression in the PVH. We used fast-refeeding conditions since it is known that insulin levels are increased in refeeding after fasting. To ensure that the potential effect on C-Fos induction is specific to insulin changes, we also included STZ-treated insulin deficient mice. As expected, compared to controls non-fasting (Ctrl N-F), fast-refeeding (Ctrl F-R) induced a significantly higher level of insulin, which was absent in STZ-treated mice (STZ N-F and F-R, Fig. 4a). Despite insulin deficiency, compared to non-fasting groups, STZ-treated group consumed a large amount of food, at a comparable level of that in control refed group (Fig. 4b), suggesting that feeding behaviors are not associated with the level of C-Fos expression in the PVH. Interestingly, fast-refeeding significantly induced C-Fos expression in the PVH in control mice while failed to do so in STZ-treated animals (Fig. 4c,d). Collectively, these results suggest that an increased insulin level contributes to C-Fos expression in the PVH in fast-refeeding conditions.

**Figure 5:**
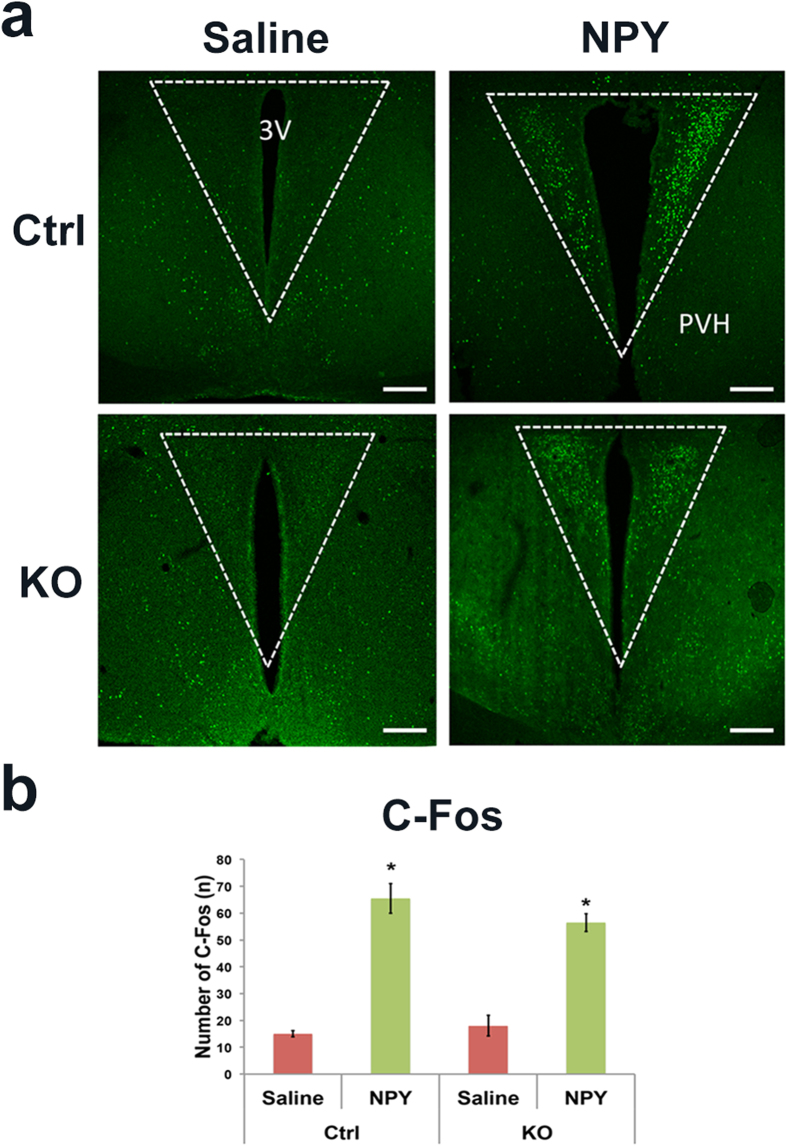
NPY induced C-Fos expression in PVH independent of GABAergic action on PVH neurons. *Sim1-Cre:γ2*^*flox/flox*^ KO mice and control mice were icv administered with saline or NPY (2 μg in 2 μl) and C-Fos expression in the PVH was examined 2 hours later. (**a**) Representative pictures showing C-Fos immunoreactive neurons in the PVH of WT (control, upper panels), KO mice (*Sim1-Cre:γ2*^*flox/flox*^, bottom panels), saline treatment (left panels) and NPY treatment (right panels). (**b**) Statistical analysis of the number of C-Fos neurons in the PVH of mice described in (**a**). Scale bar is 500 μm. 3 V: the third ventricle. Data presented as mean ± SEM, n = 5–6 mice, *p < 0.05 and **p < 0.01, two way ANOVA tests with tukey post hoc analyses.

Previous observations strongly support the notion that NPY reduces presynaptic release onto PVH neurons^20^, which may lead to activation of PVH neurons owing to “disinhibition” of reduced GABA release. To investigate the possibility that NPY induces C-Fos expression in the PVH by reduced presynaptic GABA release, we used a previously established mouse model with disruption of GABA-A receptor function in PVH neurons by deleting GABA-A receptor γ2 subunit (*Sim1-Cre:γ2*^*flox/flox*^ mice)^32^. In this model, response of PVH neurons to GABA is severely diminished^32^ and therefore the effect caused by NPY to reduce presynaptic GABA release on PVH neuron activity will be minimal. While NPY induced C-Fos expression in the PVH compared to controls (Fig. 5a), it also induced abundant C-Fos expression in the PVH in *Sim1-Cre:γ2*^*flox/flox*^ mice (Fig. 5b). Statistical analysis showed that C-Fos induction by NPY in *Sim1-Cre:γ2*^*flox/flox*^ mice was not different from that in control animals (Fig. 5c), suggesting that NPY induced C-Fos expression in the PVH is not mediated by reduced GABAergic action on PVH neurons.

## Discussion

This study was designed to address the apparent discrepancy between the hyperphagic effect of NPY and its effects on C-Fos induction in the PVH. Lesion of PVH causes hyperphagia and obesity^33^,^34^. Inactivation of PVH neurons leads to hyperphagia while activation of these neurons reduces feeding^13^,^14^,^15^, suggesting that the overall role of PVH neurons is to reduce feeding behavior. Counter-intuitively, it is well-established that NPY induces a profound feeding behavior^1^,^2^, which is accompanied with abundant C-Fos expression in the PVH^8^,^9^. The induction of C-Fos in the PVH has been suggested to be mediated by direct intracellular signaling pathways within PVH neurons through NPY receptors or indirectly through reducing presynaptic GABA release^20^,^21^,^35^. In this study we provided compelling evidence supporting that insulin action, but not GABA-A receptors, contributes significantly to NPY-induced C-Fos in the PVH. Our results showed that insulin deficiency reduced the ability of NPY to induce C-Fos in the PVH, suggesting that insulin action is required for NPY induction of C-Fos. In addition, fasting conditions, known to have low insulin levels but high NPY levels, was associated with background levels of C-Fos in the PVH while fast-refeeding condition with high insulin levels was associated significantly higher level of C-Fos expression. Consistently, fast-refeeding induced C-Fos in the PVH was absent in insulin-deficient conditions, suggesting an important role for insulin action in C-Fos induction. Importantly, our data also showed that insulin and NPY induced C-Fos expression in the same group of PHV neurons. These data, taken together, strongly support the notion that insulin action contributes to NPY-induced C-Fos in the PVH. This notion is consistent with the previous findings that central NPY action increases blood insulin levels^23^,^24^,^25^ and that, in electrophysiological recordings, NPY directly inhibits PVH neurons rather than excites them^36^. Supporting this, leptin suppresses NPY expression while induces C-Fos in the PVH^37^, and the number of PVH neurons expressing NPY receptors Y1, Y2 and Y5 in previous studies^7^,^38^ appears to be much less than that with NPY induced C-Fos expression. Of note, our current experiments were not designed to test whether C-Fos induction in the PVH by insulin is mediated directly by insulin receptors expressed in the PVH or indirectly through other brain sites. Further studies using PVH-specific loss of insulin action are warranted to address this issue.

A recent study suggests that activation of thyrotropin releasing hormone (TRH) neurons in the PVH promotes feeding by a direct excitatory projection to AgRP/NPY neurons in the arcuate nucleus^39^, raising the possibility that TRH neurons may be directly activated by NPY to mediate the NPY hyperphagia effect. Due to difficulty in labelling TRH neuron soma using TRH immunostaining, it is unknown whether NPY-induced C-Fos is located in TRH neurons. Despite this, it is unlikely that TRH neurons directly mediate the NPY hyperphagia effect. Firstly, it has been demonstrated that TRH neurons are not a direct downstream neurons of AgRP/NPY neurons^39^. Secondly, during fasting where NPY is activated, TRH neurons as well as the hypothalamic pituitary thyroid axis are known to be severely inhibited^40^, arguing against a direct activation of NPY on TRH neurons. Thirdly, it has been shown that NPY produces an inhibitory action on TRH neurons^36^.

It has been previously shown that NPY reduces presynaptic GABA release onto PVH neurons^20^, raising a possibility that NPY activates PVH neurons through “disinhibition” by removing GABAergic inhibition. Using an animal model with disrupted function of GABA-A receptors, our data showed that the degree of NPY in inducing C-Fos expression in the PVH was insensitive to defective GABA-A receptors in PVH neurons. Since PVH neurons with loss of GABA-A receptor γ2 subunit exhibit greatly diminished response in GABA-mediated inhibitory postsynaptic currents^32^,^36^, the ability of NPY to be able to reduce GABA-mediated action on PVH neurons will be minimal. Thus, our study argues strongly against a direct role of NPY on C-Fos expression by reducing GABAergic action on PVH neurons.

It is important to point out that the level of C-Fos expression in the PVH is not associated with the degree of hyperphagia induced by NPY. STZ-treated insulin deficient mice exhibited the same level of feeding as controls while showing much less number of C-Fos neurons in the PVH. Consistently, central insulin administration produced a similar induction of C-Fos neurons in the PVH, it didn’t produce any significant effects on feeding in this study (data not shown), and was even reported to reduce feeding^41^,^42^. In addition, disruption of GABA-A receptor function in the PVH leads to blunted NPY action on feeding^26^, but had no effects on NPY action in C-Fos induction in the PVH. Thus, it appears that NPY-induced C-Fos in the PVH and NPY-induced hyperphagia are two separate events. NPY-induced feeding is presumably a result, at least partly, of its directly inhibitory effects on PVH neurons. NPY-induced C-Fos in the PVH is maybe a result of actions from secondary factors owing to NPY action in the PVH or other brain sites and insulin is at least one of the secondary factors that contribute to NPY-induced C-Fos in the PVH.

In summary, our results showed that NPY-induced feeding and C-Fos expression in the PVH were two independent events and that insulin action contributed to C-Fos expression. Therefore, caution should be exercised to rely on NPY-induced C-Fos expression to study NPY feeding pathways.

## Additional Information

**How to cite this article**: Fan, S. *et al.* An Indirect Action Contributes to C-Fos Induction in Paraventricular Hypothalamic Nucleus by Neuropeptide Y. *Sci. Rep.* **6**, 19980; doi: 10.1038/srep19980 (2016).
